# Characterization of IL-21-expressing recombinant hepatitis B virus (HBV) as a therapeutic agent targeting persisting HBV infection

**DOI:** 10.7150/thno.44715

**Published:** 2020-04-25

**Authors:** Zhongliang Shen, Jingwen Wu, Zixiang Gao, Jingyu Wang, Haoxiang Zhu, Richen Mao, Xuanyi Wang, Jiming Zhang, Youhua Xie, Jing Liu

**Affiliations:** 1 Department of Infectious Diseases, Huashan Hospital, Fudan University, Shanghai 200040, People's Republic of China; 2 Key Laboratory of Medical Molecular Virology (MOE/NHC/CAMS), Department of Microbiology and Parasitology, School of Basic Medical Sciences, Shanghai Medical College, Fudan University, Shanghai 200032, People's Republic of China; 3 Key Laboratory of Medical Epigenetics and Metabolism, Institutes of Biomedical Sciences, School of Basic Medical Sciences, Shanghai Medical College, Fudan University, Shanghai 200032, People's Republic of China

**Keywords:** hepatitis B virus, interleukin 21, recombinant HBV vector, superinfection, HBV clearance

## Abstract

Chronic infection by hepatitis B virus (HBV) is associated with high risks of liver fibrosis, cirrhosis and hepatocellular carcinoma. In mouse models of HBV persistence, interleukin 21 (IL-21) has been identified as a potent inducer of viral clearance. Strict hepatotropism makes recombinant HBV (rHBV) vectors ideal for liver-targeting gene delivery. Previously, we established an rHBV vector termed 5c3c, which is highly replicative by itself, but requires HBV envelope proteins provided *in trans* to produce virions. 5c3c-based rHBV virions are capable of delivering cargo gene expression driven by HBV Sp1 promoter into infected hepatocytes. In this work, we explore the feasibility of using 5c3c-derived rHBV for liver-specific delivery of IL-21 as treatment of chronic HBV infection.

**Methods:** 5c3c-derived rHBV replicons harboring mouse or human IL-21 genes (termed 5c3c-mIL-21 and 5c3c-hIL-21 respectively) were constructed and then tested for the production of rHBV virions* in vitro* and *in vivo*. 5c3c-mIL-21's anti-HBV effects were determined in chronic HBV mouse model. Furthermore, superinfection by rHBV virions was analysed using HBV-infected HepG2/NTCP cells and human liver chimeric mice.

**Results:** 5c3c-mIL-21 and 5c3c-hIL-21 were efficiently replicative and produced enveloped virions when provided with envelope proteins, both *in vitro* and *in vivo*. In mouse model of HBV persistence, IL-21 expressed from injected 5c3c-mIL-21 replicon induced complete viral clearance. 5c3c-mIL-21 and 5c3c-hIL-21 virions could infect HepG2/NTCP cells and engender sustained IL-21 expression. Most importantly, IL-21-expressing rHBV virions could superinfect HBV-infected HepG2/NTCP cells and human hepatocytes in human liver chimeric mice, and engender sustained IL-21 expression and rHBV production.

**Conclusion:** These data suggest the high potential of 5c3c-derived IL-21-expressing rHBV as a novel therapeutic against chronic HBV infection.

## Introduction

Hepatitis B virus (HBV) chronically infects ~250 million people worldwide 1. As a risk factor for liver fibrosis, cirrhosis and hepatocellular carcinoma, chronic HBV infection is associated with ~0.88 million deaths annually 1. Current treatment options for chronic HBV infections (CHB), interferon α and especially nucleos(t)ide analogues, have been useful in controlling virus activity but relatively ineffective in inducing viral clearance 2. HBV takes humans as natural hosts and exclusively infects hepatocytes *in vivo* 3, 4. HBV virion contains a ~3.2 kb partially double-stranded relaxed circular DNA (rcDNA) genome that is converted into covalently closed circular DNA (cccDNA) in the nucleus of infected cell. HBV cccDNA is the sole template of viral transcription, and 4 viral promoters (Cp, Sp1, Sp2, and Xp) drive the production of at least 4 RNA species of 3.5, 2.4, 2.1, and 0.7 kb in length, respectively. HBV polymerase (P), translated from 3.5 kb pregenomic RNA (pgRNA), binds to pgRNA *in cis* to form polymerase-pgRNA complex to initiate reverse transcription. The complex is then packaged by core proteins (HBcAg), also translated from pgRNA, to form progeny nucleocapsid, wherein reverse transcription continues and rcDNA is eventually produced. Nucleocapsids could be enveloped by viral large/middle/small (L/M/S) surface or envelop proteins (HBsAg) to form mature virions, or recycled back to nucleus to form new nuclear cccDNA 3. Neither interferon α nor nucleos(t)ide analogues affect cccDNA 2, 5. It has also been observed that HBV cccDNA stably persists in infected hepatocytes regardless of the outcome of antiviral treatment, and may be reactivated in patients after 'successful' antiviral therapy 6.

Previously, by using multiple mouse models of HBV persistence, we identified and confirmed interleukin 21 (IL-21) as a potent inducer of HBV clearance 7, 8. We demonstrated that liver-targeted delivery of IL-21 expression using plasmid DNA 8 or recombinant adeno-associated virus (AAV) 7 effectively clears persisting HBV DNA, including both replicon plasmid DNA and cccDNA mimic DNA, from mouse liver. These data supported IL-21 as a valid candidate for developing novel CHB therapeutics. However, due to the pluripotency of IL-21 as an immune regulator 9, 10, the delivery method requires further investigation and optimization in order to reduce or avoid unwanted side effects. On one hand, liver-targeting injection of plasmid DNA is unlikely to be applicable to human; on the other, serotype(s) of AAV that display preferential transduction of hepatocytes are not exclusively liver-specific.

Despite many restrictions imposed by its complex and compact genome organization, HBV's strict hepatotropism makes it an ideal vector for liver- targeting gene delivery 11. Previously, we generated a highly replicative recombinant HBV (rHBV) vector 5c3c that harbors a 384 base pairs (bp) deletion in the polymerase spacer region for cargo gene insertion 12. 5c3c requires wild type HBV envelope proteins provided *in trans* to be able to produce mature virions, but its genome replication is self-reliant. 5c3c-derived virions are infective for primary tupaia hepatocytes and engenders cargo gene expression in infected cells 12. In this work, we explored the possibility of using 5c3c as vector for hepatocyte-specific IL-21 delivery. Our results show that 5c3c-based rHBV replicons carrying mouse or human IL-21 coding sequences under the control of HBV Sp1 promoter were sufficiently replicative in transfected cells and produced enveloped virions in the presence of co-transfected envelope proteins. Mouse IL-21 expressed from rHBV replicon plasmid that was delivered into mice via hydrodynamic injection (HDI), was effective in inducing HBV clearance in mice harboring HBV persistence. IL-21-harboring rHBV virions could infect HepG2 cells stably expressing HBV receptor NTCP (HepG2/ NTCP), which resulted in sustained IL-21 expression. Most importantly, such virions were capable of super-infecting both HepG2/NTCP cells previously infected with wild type HBV, and hepatocytes in human liver chimeric mice previously infected with wild type HBV, and sustained rHBV production and cargo gene expression by superinfected cells was observed. These results demonstrated 5c3c-derived rHBV virions as promising liver-specific IL-21- delivering vectors that could prove useful for CHB treatment.

## Results

### Adeno-associated virus (AAV)-mediated gene delivery lacks strict liver-tropism in mice

Previously, we demonstrated that IL-21 delivered by recombinant serotype 8 AAV virus elicited clearance of HBV persistence in mouse models 7. Among AAV serotypes commonly tested for gene therapy, serotype 8 is generally accepted to possess higher liver-tropism 13, 14. However, in BALB/c mice injected with serotype 8 AAV expressing EGFP (AAV-EGFP) via tail vein, in addition to prominent EGFP expression in liver as expected, EFGP expression was also observed in heart and, to a lesser extent, in kidney (**Figure S1**). This is consistent with previously published data 14, 15. Clearly, AAV vectors are incapable of transducing with strict liver-tropism, and as therapeutic vector for treating chronic HBV infection, which is exclusively hepatocyte-specific, may cause unwanted effects due to extra-liver transductions. In this regard, recombinant HBV (rHBV) vectors constitutes a more preferable alternative since they have the same tissue-tropism as wild type HBV.

### Construction and preparation of recombinant HBV expressing IL-21

In order to evaluate rHBV as vector for liver-specific delivery of IL-21, we used our previously described rHBV vector 5c3c 12. 5c3c harbors a maximized in-frame deletion of 384 bp within the functionally non-critical spacer region of polymerase, where cargo gene sequences can be inserted in-frame with the overlapping preS1-preS2-S ORF. Inserted sequences need to be synonymously mutated wherever necessary to avoid introducing termination codon in the overlapping polymerase reading frame, and expression of cargo gene is driven by the upstream HBV preS1 promoter. 5c3c-derived rHBV replicons generally retain replication efficiency and produce infective rHBV virions in the presence of HBV envelope proteins expressed from helper plasmid or wild type virus 3.

**Figure 1:**
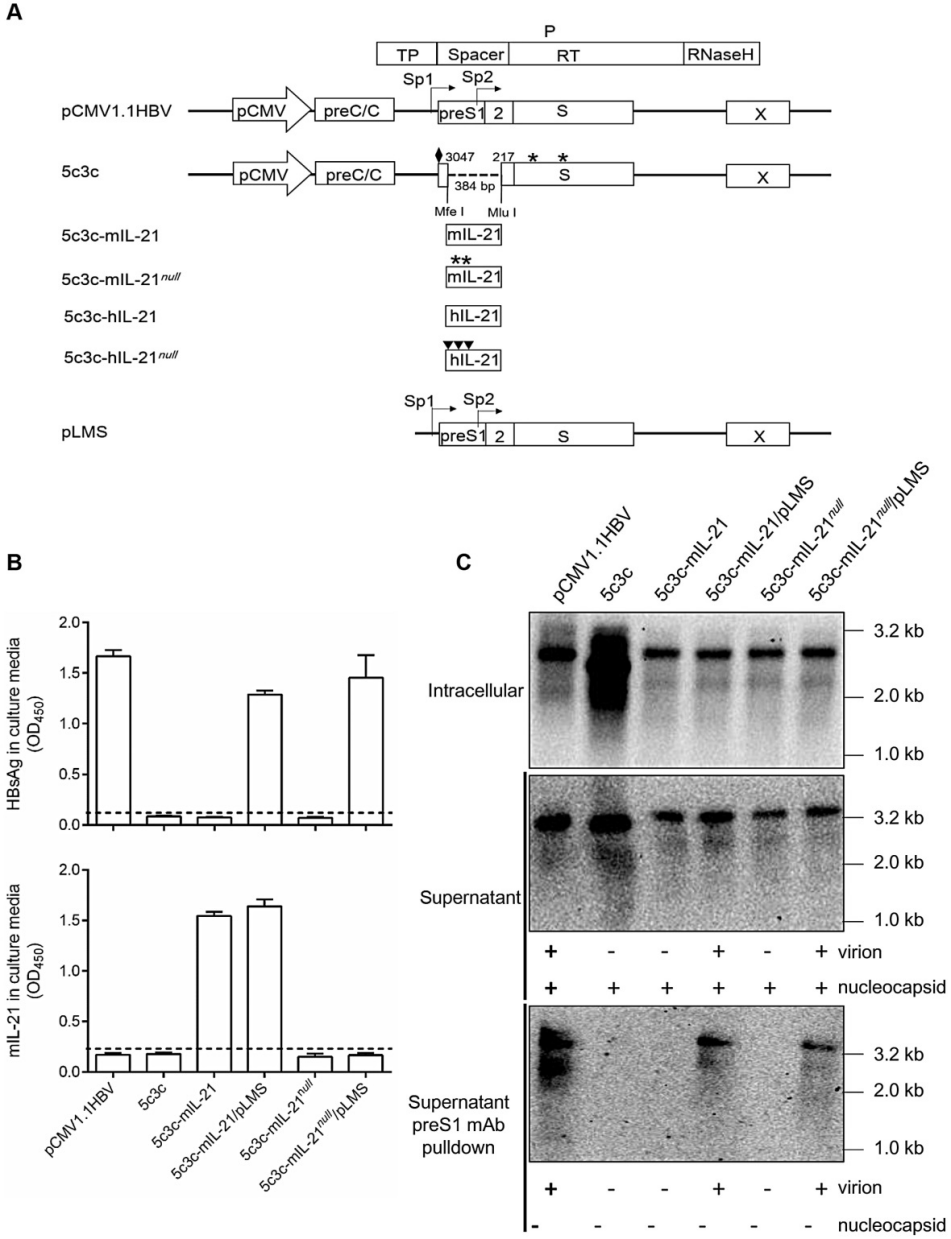
** Construction and characterization of 5c3c-derived rHBV expressing IL-21.** (**A**) Schematic representation of plasmid constructs. Open boxes and arrows represent ORFs and promoters, respectively. Stop codons in the S and murine IL-21 (mIL-21) ORFs are indicated by asterisks. Solid diamonds indicate ATG to ACG mutation at preS1 start codon. Solid triangles indicate ATG to ACG mutation at 1^st^, 8^th^ and 11^th^ codons in human IL-21 (hIL-21) ORF. Restriction enzyme sites (*Mfe*Ⅰ and *Mlu*Ⅰ) used for inserting cargo sequences and nucleotide positions on HBV genome are indicated. Murine and human IL-21 sequence modifications are detailed in **Figure S2** and **Figure S3**. (**B**) Supernatants from Huh-7 transfected with indicated plasmids in triplicates were assayed for HBsAg (top) and mIL-21 (bottom) using ELISA. Group means and SEMs are presented. Dotted lines represent cut-off thresholds. (**C**) Southern blot analysis of intracellular capsid-associated HBV DNA (top), capsid- and virion-associated HBV DNA in the supernatants (middle), and virion-associated DNA in the supernatants captured by anti-preS1 mAb (bottom). Expected presence of HBV capsid and virions in supernatants with or without anti-preS1-capture are indicated below the blots. Transfections were independently repeated with similar results and representative data from one experiment are shown.

Murine IL-21 coding sequences (441 bp) modified according to the above rule were inserted into 5c3c to create 5c3c-mIL-21, and premature termination was introduced in mIL-21 reading frame to create 5c3c-mIL-21*^null^* as IL-21-deficient control (**Figure S2** and** Figure 1A**). 5c3c-mIL-21 and 5c3c-mIL-21*^null^* have the same genome size of 3287 bp, compared to 2846 bp of 5c3c and 3215 bp of wild type. When the respective replicon plasmids were transfected into Huh-7 cells, expectedly only 5c3c-mIL-21 produced measurable mIL-21 in culture supernatants (**Figure 1B**), while both 5c3c-mIL-21 and 5c3c-mIL-21*^null^* displayed replication efficiencies comparable to wild type HBV (**Figure 1C**). Similar results were obtained when human IL-21 (hIL-21) coding sequences (405 bp) were used (**Figure S3** and**Figure S4**). Furthermore, when and only when co-transfected with helper plasmid expressing HBV envelope proteins (pLMS), both 5c3c-mIL-21 and 5c3c-mIL-21*^null^* secreted enveloped virions into culture media (**Figure 1C**). These data indicated that 5c3c- based rHBV replicon harboring mIL-21 cargo gene could support mIL-21 expression in transfected cells of liver origin, and package mature rHBV virions in the presence of HBV envelope proteins. Viral DNA was also detectable in supernatants of 5c3c-mIL-21- and 5c3c-mIL-21*^null^*-transfected cells in the absence of envelope proteins (**Figure 1C**), indicating secretion of unenveloped viral nucleocapsids by replicon-transfected cells as confirmed by anti-HBcAg antibody pulldown followed by Southern blot analysis (**Figure S5**).

**Figure 2:**
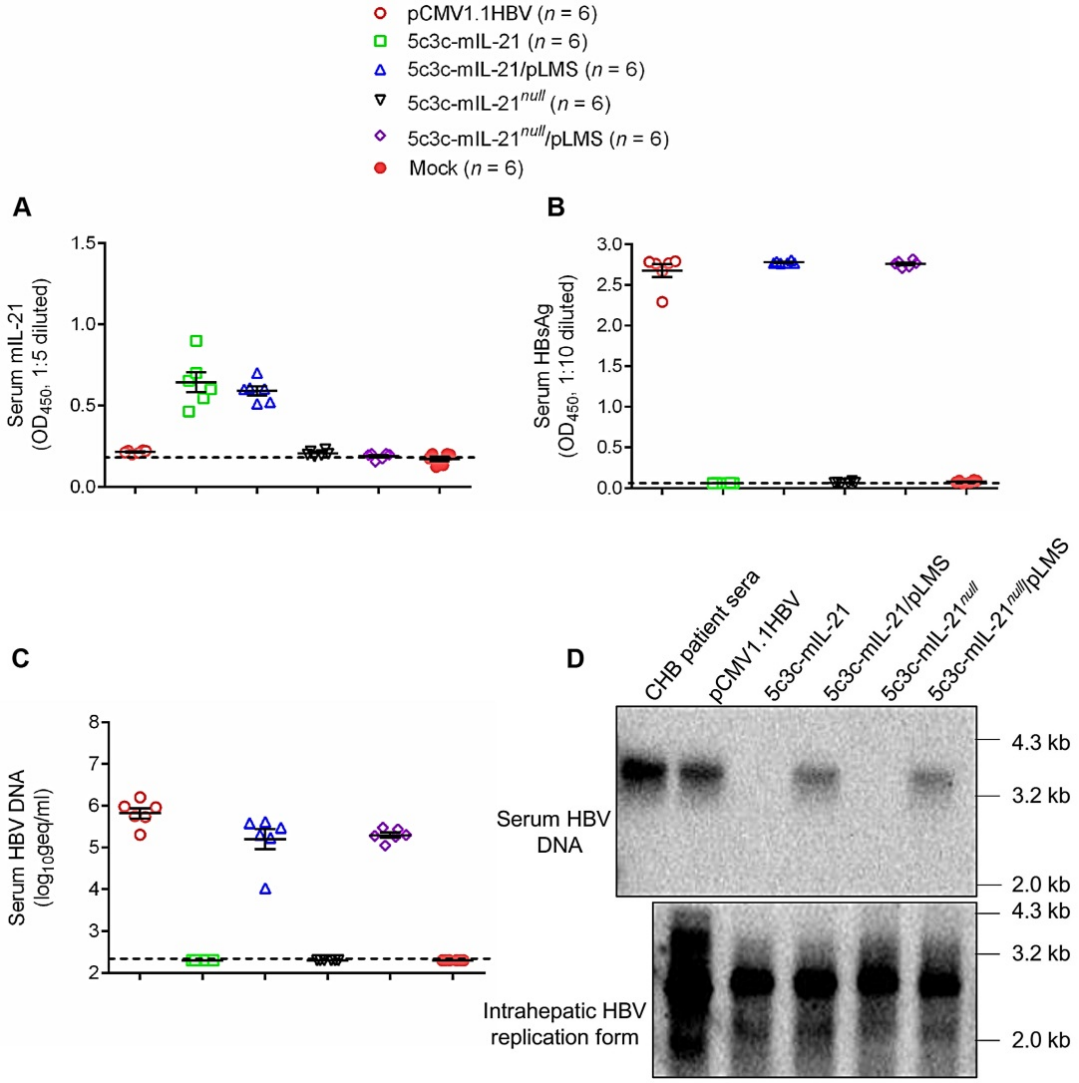
** Production of IL-21-expressing rHBV virions in mice.** BLAB/c mice were injected with 10 μg each of indicated plasmid(s) using hydrodynamic injection via tail vein or left untreated. Sera were collected 3 days later and assayed for mIL-21 (**A**) and HBsAg (**B**) using ELISA, and HBV DNA using quantitative real time PCR (**C**). Southern blot was performed to analyze HBV DNA present in pooled mouse sera, and HBV replication intermediates present in mouse liver tissue (**D**). Sera from chronic hepatitis B (CHB) patients were used as control. Dotted lines represent cut-off thresholds (**A** and **B**), and lower limit of quantification (**C**), respectively. Group means and SEM within group are presented with group sizes (*n*) indicated. geq, genome equivalents.

Genome replication and progeny virus production capabilities of 5c3c-derived IL-21-expressing rHBV replicons were then further tested *in vivo* after hydrodynamic injection (HDI) into BALB/c mice via tail vein with or without helper plasmid. As shown in **Figure 2**, HDI with 5c3c-mIL-21 replicon resulted in markedly elevated serum levels of IL-21. Interestingly, serum 5c3c-mIL-21 rHBV DNA was only detectable in mice co-injected with helper plasmid, but not in mice injected with replicon plasmid alone, despite the fact that Southern blot analysis of intracellular capsid-associated HBV DNA showed that both 5c3c-mIL-21 and 5c3c-mIL-21*^null^* replicons were efficiently replicative (**Figure 1**). Apparently, unenveloped nucleocapsids observed in culture media of replicon-transfected cells (**Figure 1C**) were not present in serum from mice injected with the same replicon. In human infections, such unenveloped nucleocapsids were not detectable in serum either 12. These data not only showed that 5c3c-mIL-21 could replicate and, in the presence of envelope proteins, produce progeny rHBV virions in hepatocytes, but also illustrate an interesting and important difference between *in vitro* and *in vivo* HBV replication systems. Since rHBV virions produced in HDI mice are not contaminated with unenveloped nucleocapsids, further separation and purification are not required, which simplifies rHBV production.

### IL-21 expressed from rHBV replicon cleared persisting HBV from mouse liver

**Figure 3:**
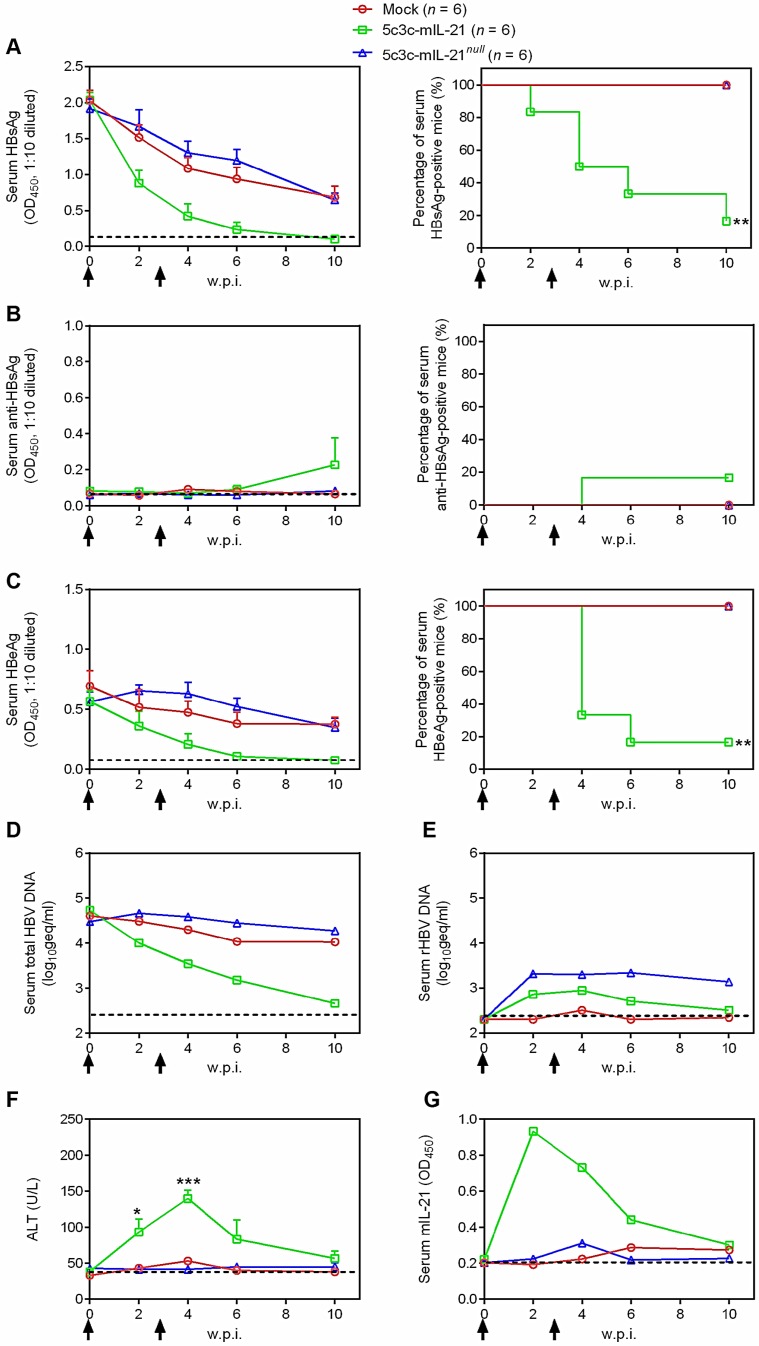
** mIL-21-expressing rHBV replicon induces viral clearance in HBV persistence mice.** BALB/c mice injected with BPS replicon plasmid using hydrodynamic injection (HDI) via tail vein that remained persistently positive for serum HBV antigens for 4 weeks were injected using HDI twice (solid arrows), at a 3-week interval, with 25 μg of 5c3c-mIL-21 or 5c3c-mIL-21*^null^*, or left untreated. Sera were collected at indicated time points and analyzed for HBsAg (**A**), HBsAb (**B**), HBeAg (**C**), total HBV DNA (pooled sera, **D**), rHBV DNA (pooled sera, **E**), ALT (**F**) and murine IL-21 (pooled sera, **G**). Group means and SEM within group are presented with group sizes (*n*) indicated. Dotted lines represent cut-off thresholds (**A**, **B** and **C**, left panels, **E**) pre-injection baseline (**D**) or lower limit of quantification (**F** and **G**), respectively. Dotted lines represent cut-off thresholds (**A**, **B** and **C**, left panels, **G**), lower limit of quantification (**D** and **E**) or pre-injection baseline (**F**), respectively. Data from 5c3c-mIL-IL-21 and 5c3c-mIL-21*^null^* groups are compared against untreated group and statistical significance calculated using log-rank (Mantel-Cox) test (**A**, **B** and **C**, right panels) or unpaired two-tailed t-test (**F**). *, *P* < 0.05; **, *P* < 0.01; ***, *P* < 0.001. w.p.i., weeks post first injection of rHBV replicon. geq, genome equivalents.

In our previous studies, CMV promoter-driven expression of IL-21 from plasmid or recombinant AAV induced viral clearance in BALB/c mice harboring HBV (genotype B strain BPS) persistence 7, 8. To test whether IL-21 expression driven by HBV promoter is as effective in inducing clearance, mice harboring pre-established BPS persistence (serum HBV antigens persistently positive for at least 4 weeks) were given two HDI injections, 3 weeks apart, of 5c3c-mIL-21 or 5c3c-mIL-21*^null^* replicon plasmids. As shown in **Figure 3**, injection of 5c3c-mIL-21, but not 5c3c-mIL-21*^null^*, resulted in markedly accelerated decrease of serum HBsAg and HBeAg in all recipient mice, and eventual disappearance of both markers in 5 out of 6 mice by 10 weeks post first injection (w.p.i.) of rHBV replicon plasmid. Similarly, HBV DNA in pooled sera started to decrease rapidly in 5c3c-mIL-21-injected mice after the first injection and dropped close to detection limit by 10 w.p.i. (**Figure 3D**). In contrast, decrease of serum HBsAg and HBeAg was comparably slow in 5c3c-mIL-21*^ null^*-injected and untreated mice, and none of these mice cleared any viral marker by 10 w.p.i., with serum HBV DNA levels remaining largely unchanged through the observation period (**Figure 3A-D**).

Since BPS expresses both large and small envelope proteins that support packaging of mature, infective virions 8, we also investigated the dynamics of rHBV production in BPS persistence mice receiving rHBV replicon injections by using quantitative real time PCR targeting rHBV-specific sequences. As shown in **Figure 3E**, rHBV DNA was readily detectable in serum after 5c3c-mIL-21 or 5c3c-mIL-21*^null^* replicon injection and remained at nearly unchanged levels till 4 w.p.i.. Afterwards, rHBV DNA levels started to decrease and dropped to near detection limit by 10 w.p.i. in 5c3c-mIL-21-injected mice. In 5c3c-mIL-21*^ null^*-injected mice, however, rHBV DNA levels did not change markedly up till the end of observation period (**Figure 3E**), similar to what was observed for total HBV DNA (**Figure 3D**).

In order to confirm the clearance of BPS in 5c3c-mIL-21-injected mice, mouse liver DNA was extracted at 10 w.p.i. and subjected to Southern blot analysis using BPS- and rHBV-specific probes. As shown in **Figure S6**, in 5c3c-mIL-21-injected BPS persistence mice that cleared serum HBsAg at 10 w.p.i., neither BPS nor 5c3c-mIL-21 replicon DNA was detectable in liver; whereas in 5c3c-mIL-21*^null^*-injected mice, which failed to clear serum HBsAg, both BPS and 5c3c-mIL-21*^null^* DNA persisted at considerable levels. These results are in agreement with serum viral DNA analysis results (**Figure 3D-E**) and proved true and complete clearance of both wild type BPS and rHBV DNA in 5c3c-mIL-21-injected BPS persistence mice.

Mechanistically, 5c3c-mIL-21-induced HBV clearance was not associated with HBsAb development (**Figure 3B**), but coincided with transient ALT elevations peaking at 4 w.p.i. and reverting to normal levels by 10 w.p.i. (**Figure 3F**), which was slightly preceded by a transient serum IL-21 elevations peaking at 2 w.p.i. (**Figure 3G**). When cellular immune response at 10 w.p.i. were analyzed, splenocytes from 5c3c-mIL-21-treated mice contained more HBsAg- and HBcAg-specific IFN-γ-producing cells compared to untreated and 5c3c-mIL-21*^null^*-treated mice (**Figure S7**). These data are consistent with our previous studies using plasmid and recombinant AAV that expressed IL-21 under the control of CMV promoter 7, 8, and demonstrated that 5c3c-mIL-21-mediated IL-21 expression is equally effective in inducing clearance in BPS mice.

### IL-21-expressing rHBV virions are infective* in vitro*

**Figure 4:**
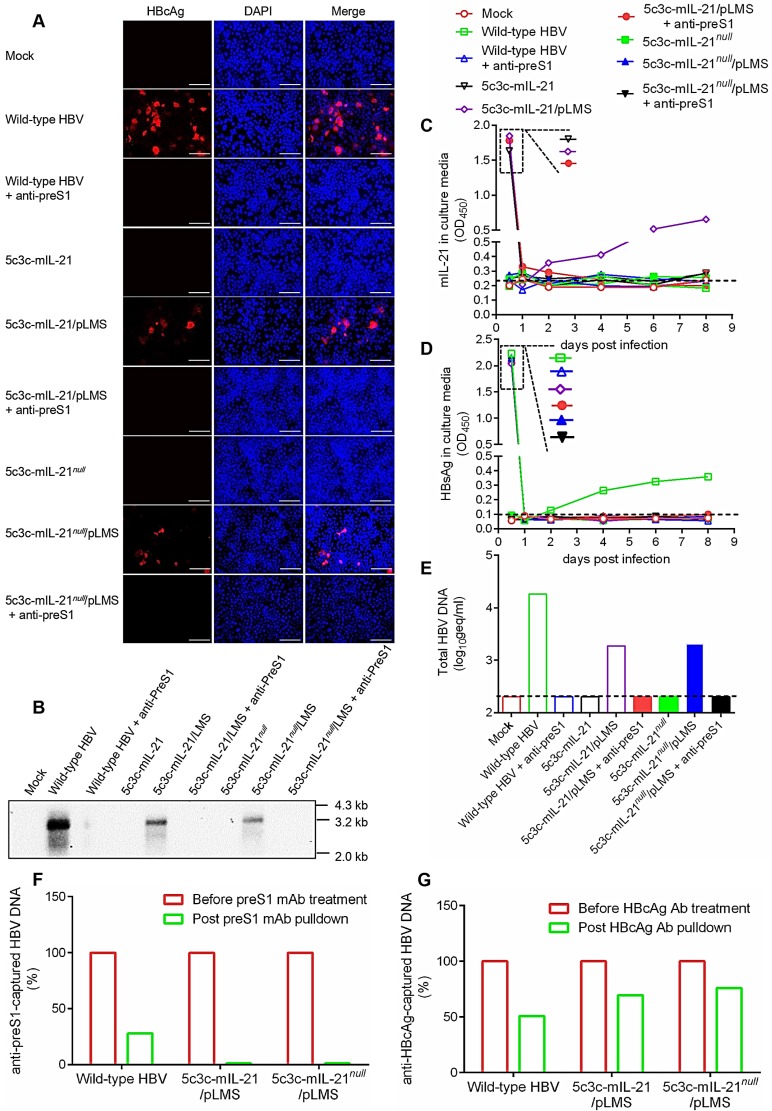
** Infection of HepG2/NTCP cells by mIL-21-expressing rHBV virions.** Concentrated supernatants from HepAD38 cells, 5c3c-mIL-21/pLMS and 5c3c-mIL-21*^null^*/pLMS co-transfected Huh-7 cells, with or without pre-incubation with anti-preS1 mAb, were used to infect HepG2/NTCP cells. Concentrated supernatants from mock transfection was used as negative control. Concentrated supernatants from 5c3c-mIL-21 and 5c3c-mIL-21*^null^* mono-transfections, which contained only naked nucleocapsids (see **Figure 1**), were used to exclude the possibility of capsid-mediated transduction. Culture media were changed at day 0.5, 1 and 2 post infection, and then every 2 days. On day 8, cells were subjected to DAPI and anti-HBcAg staining and visualized using fluorescence microscopy (**A**), and intracellualr HBV replication was analyzed in Southern blot using HBV-specific probes (**B**). Scale bars, 100 μm. mIL-21 (**C**) and HBsAg (**D**) in culture media at indicated time points were measured using ELISA. (**E**) HBV DNA in day 8 culture media was analyzed using quantitative real time PCR. (**F**) HBV DNA in day 8 culture media of cells infected with indicated supernatants were analyzed using quantitative real time PCR with or without capture by anti-preS1 mAb or anti-HBcAg antibody. Captured HBV DNA levels were normalized against total HBV DNA levels without antibody pulldown. Dotted lines represent cut-off thresholds (**C** and **D**), and lower limit of quantification (**E**), respectively. ELISA and qrtPCR were performed in duplicates and triplicates, respectively, and mean values were used for plotting. Infections were independently repeated with similar results and representative data from one experiment are shown.

In order to qualify for therapeutic applications in human, infectivity of IL-21-expressing rHBV virions needs to be validated first. For this purpose, HepG2 cells stably transfected with HBV receptor NTCP (HepG2/NTCP) were incubated with 5c3c-mIL-21/pLMS or 5c3c-mIL-IL-21*^null^*/pLMS co-transfection supernatants, or supernatant from HepAD38 cells as wild type HBV control. As shown in **Figure 4A**, immunofluorescence staining readily detected intracellular HBcAg in HepG2/NTCP cells infected with HepaAD38 supernatant, or 5c3c-mIL-21/pLMS or 5c3c-mIL-IL-21*^null^*/pLMS co-transfection supernatants. No HBcAg was detectable in cells incubated with 5c3c-mIL-21 or 5c3c-mIL-IL-21*^null^* mono-transfection supernatants, which reflected envelope-dependent morphogenesis and infectivity of rHBV virions. Furthermore, when transfection supernatants were pre-incubated with anti-preS1 mAb prior to infection, no intracellular HBcAg was detectable anymore in cells infected with HepaAD38 supernatant, or 5c3c-mIL-21/pLMS or 5c3c-mIL-IL-21*^null^*/ pLMS co-transfection supernatants (**Figure 4A**), which reconfirmed that both wild type and recombinant HBV virions enter susceptible cells through preS1-dependent mechanism(s). Similarly, Southern blot analysis showed that post-infection replication of wild type or recombinant HBV genomes were only detectable in cells infected with HepaAD38 supernatant, or 5c3c-mIL-21/pLMS or 5c3c-mIL-IL-21*^null^*/pLMS co-transfection supernatants (**Figure 4B**).

Culture supernatants of infected HepG2/NTCP cells were also serially sampled and analyzed. As shown in **Figure 4C**, sustained and increasing mIL-21 expression and secretion by rHBV was only detected in supernatants from cells infected with 5c3c-mIL-21/ pLMS co-transfection supernatant, whereas sustained and increasing HBsAg secretion was only detectable in supernatants from cells infected with wild type HBV (**Figure 4D**). These results are consistent with the expected IL-21 and HBsAg expression capacities of the respective virions (**Figure 1** and**Figure S2**). In addition, when HBV DNA in culture supernatants were analyzed at the end of observation period (8 days post infection), only cells infected with HepaAD38 supernatant produced enveloped HBV DNA, namely virions, into culture media, while cells infected with 5c3c-mIL-21/pLMS or 5c3c-mIL-IL-21*^null^*/pLMS co-transfection supernatants only produced unenveloped viral DNA in the form of unenveloped nucleocapsids (**Figure 4E-F**) as observed in replicon transfection experiments (**Figure 1C**). These data are also consistent with the inability of these 5c3c-derived rHBV replicons to produce virions by themselves due to lack of envelope expression (**Figure 1** and**Figure S2**).

Highly similar results were obtained when rHBV expressing human IL-21 was used in the same assay (**Figure S8**). Moreover, 5c3c-mIL-21 and 5c3c-mIL-IL-21*^null^* rHBV virions produced in HDI mice (**Figure 2**) also displayed infectivity using this assay (**Figure S9**).

### IL-21-expressing rHBV virions superinfected HBV-infected cells *in vitro* and *in vivo*

**Figure 5:**
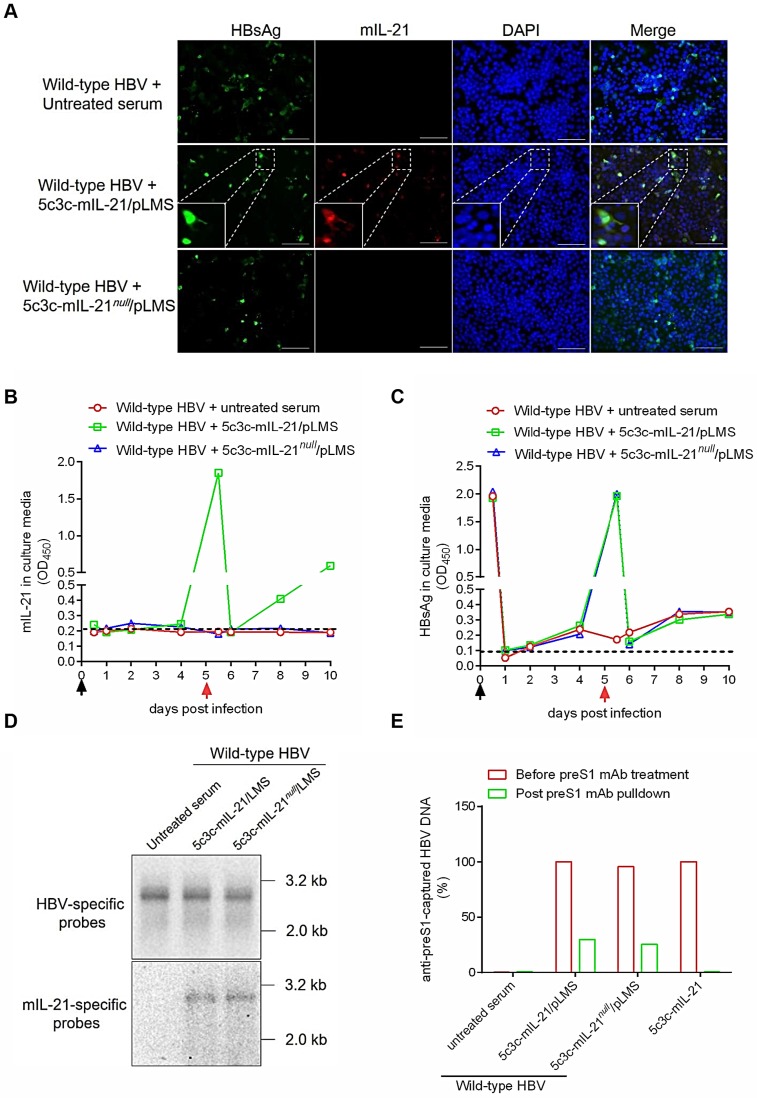
** Superinfection of HBV-infected HepG2/NTCP cells by mIL-21-expressing rHBV virions.** HepG2/NTCP cells were first infected with wild type HBV using concentrated sera from pCMV1.1HBV injected mice (black arrows). Culture media were changed at 0.5, 1, 2 and 4 days post infection (d.p.i.). At 5 d.p.i., concentrated sera from naïve, 5c3c-mIL-21/pLMS or 5c3c-mIL-21*^null^*/pLMS co-injected mice were used to superinfect HepG2/NTCP cells (red arrows). Culture media were changed on 5.5 and 6 d.p.i., and then every 2 days. (**A**) At 10 d.p.i., cells were subjected to DAPI, anti-HBsAg and anti-mIL-21 staining and visualized using fluorescence microscopy. Scale bars, 100 μm. mIL-21(**B**) and HBsAg (**C**) in culture media at indicated time points were measured using ELISA. (**D**) Wlid-type HBV and rHBV DNA replication in 10 d.p.i. cells were analyzed in Southern blot using HBV- and mIL-21-specific probes, respectively. (**E**) rHBV DNA in culture media of cells infected with indicated supernatants at 10 d.p.i. were analyzed using quantitative realtime PCR before and after capture by anti-preS1 mAb or anti-HBcAg (shown in **Figure S10**). Captured rHBV virion DNA levels were normalized against rHBV DNA levels without antibody pulldown. Supernatants from 5c3c-mIL-21 monotransfected cells, containing only non-enveloped nucelocapsids, were used as virion-free control. Dotted lines represent cut-off thresholds (**B** and** C**). ELISA and qrtPCR were performed in duplicates and triplicates, respectively, and mean values were used for plotting. Infections and superinfections were independently repeated with similar results and representative data from one experiment are shown.

For therapeutic applications targeting chronic HBV infections, IL-21-expressing rHBV should not only be infective for hepatocytes, but also be capable of superinfecting hepatocytes already infected with wild type HBV, since virtually all hepatocytes are already HBV-infected in this context 16. To test such superinfection *in vitro*, HepG2/NTCP cells were first infected with wild type HBV by incubation with sera from mice injected with wild type replicon plasmid pCMV1.1HBV via HDI, and 5 days later, incubated with sera form untreated, 5c3c-mIL-21/pLMS or 5c3c-mIL-21*^null^*/pLMS co-injected mice for superinfection. As shown in **Figure 5A**, intracellular mIL-21 could be detected in cells re-incubated with 5c3c-mIL-21 virions using immunofluorescence 5 days later, and many of the mIL-21-positive cells were also positive for HBsAg. Since 5c3c-mIL-21 does not encode HBsAg, this indicated superinfection of previously HBV-infected cells by IL-21-expressing rHBV. Analysis of serial culture supernatant samples also confirmed sustained and increasing mIL-21 production only by cells superinfected by 5c3c-mIL-21 virions (**Figure 5B**), while sustained and increasing HBsAg secretion was observed for both cells infected with wild type HBV, or superinfected with 5c3c-mIL- 21 or 5c3c-mIL-21*^null^* virions (**Figure 5C**). Southern blot using HBV- and mIL-21-specific probes in parallel confirmed rHBV replication in superinfected cells (**Figure 5D**). Moreover, both unenveloped and enveloped rHBV DNA was detectable in supernatants from cells superinfected with 5c3c-mIL-21 or 5c3c-mIL-21*^null^* virions, but not in supernatant from cells mono-transfected with 5c3c-mIL-21 only (**Figure 5E and S10**). These results demonstrated that superinfection of HBV-infected cells by 5c3c-mIL-21-derived rHBV virions not only resulted in continuous mIL-21 production, but also sustained progeny rHBV production with the help of envelope proteins produced by pre-existing wild type HBV.

**Figure 6:**
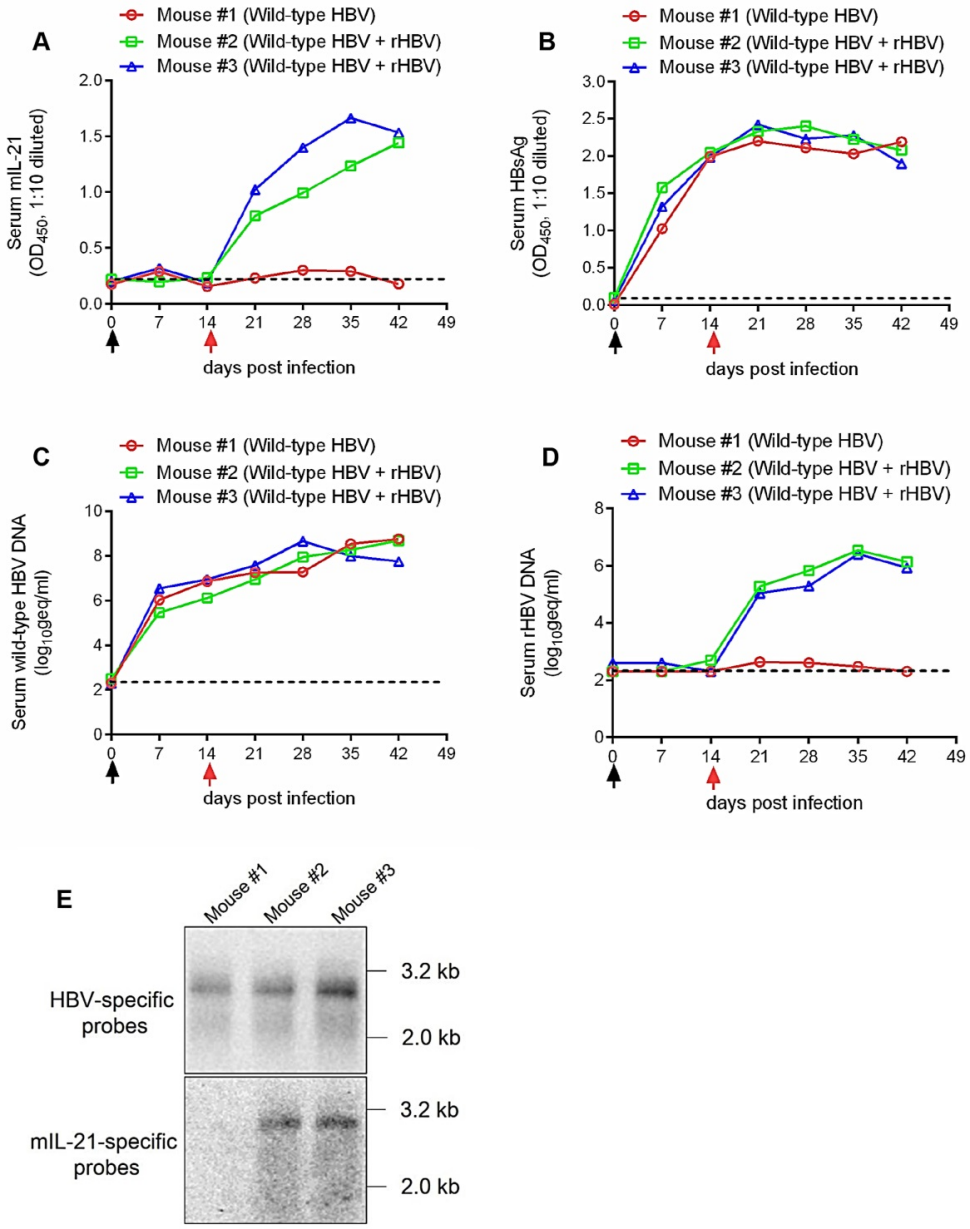
** Superinfection of HBV-infected human hepatocytes in human liver chimeric mice by mIL-21-expressing rHBV.** Human liver chimeric mice were first infected with wild type HBV by injection using concentrated sera from pCMV1.1HBV injected mice (black arrows) via tail vein. At 14 days post infection (d.p.i.), mice were injected with concentrated sea from 5c3c-mIL-21/pLMS co-injected mice (red arrows), or left untreated. Sera were collected at indicated time points. Serum mIL-21 (**A**) and HBsAg (**B**) were analyzed using ELISA. Serum wild type HBV DNA (**C**) and rHBV DNA (**D**) at indicated time points were assayed using quantitative real time PCR. At 42 d.p.i., intrahepatic wild-type HBV and rHBV replication were analyzed in Southern blot using HBV- and mIL-21-specific probes, respectively (**E**). Dotted lines represent cut-off thresholds (**A** and** B**) and lower limit of quantification (**C** and** D**), respectively. geq, genome equivalents.

Finally, human liver chimeric mice 17 were used to test rHBV superinfection *in vivo*. Mice were first infected with wild-type HBV by injection via tail vein with sera from pCMV1.1HBV HDI mice, and 14 days later, injected again with sera form 5c3c-mIL-21/ pLMS co-injected mice, or left untreated. As shown in **Figure 6A**, prominent and increasing production of mIL-21 into serum was detectable only in mice receiving 5c3c-mIL-21-derived rHBV virions. When serum HBsAg and wild type HBV DNA were measured using ELISA and quantitative real time PCR respectively, continuously increasing levels were observed in all mice (**Figure 6B-C**). In contrast, serum rHBV DNA was only detected in HBV-infected mice receiving 5c3c-mIL-21 virions (**Figure 6D**), and the levels gradually increased, in correlation with serum mIL-21 levels in the same mice (**Figure 6A**). The identity of rHBV DNA was also confirmed in Southern blot using HBV- and mIL-21-specific probes in parallel (**Figure 6E**). Collectively, these results corroborated the *in vitro* data and proved that 5c3c-mIL-21 rHBV virions are capable of superinfecting HBV-infected hepatocytes *in vivo*. Since unenveloped nucleocapsids are not observed in mice (**Figure 1D** and** Figure 5D**), increasing serum rHBV DNA levels in mice superinfected with 5c3c-mIL-21 rHBV represent marked and steady production of progeny rHBV virions by hepatocytes harboring both wild type and 5c3c-mIL-21 rHBV infection.

Tissue distribution of HBV and rHBV infection in human liver chimeric mice was evaluated after sacrifice, and as can be expected, HBV infection was only detected in liver samples and rHBV infection was only detected in liver samples from super-infected chimeric mice (**Figure S11**).

## Discussion

The potency of IL-21 for inducing viral clearance in mouse models of HBV persistence has been repeatedly demonstrated 7, 8. For potential therapeutic applications in human, however, the delivery method requires further investigation and optimization. One of the reasons for this is that given the multiple and varied functions of IL-21 9, 10, prolonged and untargeted systemic administration might not only be uneconomic, but also cause significant side effects as observed in interferon α treatment of CHB 5. Compared to protein-based methods, gene delivery methods generally require much fewer injections at significantly lower cost. Previously, we showed that recombinant AAV is capable of delivering IL-21 cargo gene into mice harboring HBV persistence and inducing viral clearance 7. The shortcomings of AAV vectors, however, are at least two-fold. Firstly, even with AAV serotypes that display preferential hepatocyte transduction, there is no strict liver-specificity (**Figure S1**). Secondly, integration of AAV genome into host chromosome remains a possibility 13. Although our limited testing did not reveal indications of severe acute extra-liver pathologies in HBV mice receiving IL-21-expressing AAV 7, results shown in **Figure S1** nevertheless would be troubling when applications in human are considered.

HBV boasts nearly exclusive hepatotropism, which even dwarfs other hepatitis viruses. Despite its small genome size and complex genome organization, our previous efforts successfully established a recombinant HBV vector system capable of delivering cargo gene expression controlled by liver-specific HBV Sp1 promoter into hepatocytes 12. Results presented in this work demonstrate that IL-21- expressing rHBV replicons are capable of replication by itself and production of mature enveloped rHBV virions using envelope proteins provided *in trans* (**Figure 1** and** Figure 2**). In addition, Sp1-driven expression of IL-21 from rHBV replicon induced viral clearance in mouse model of HBV persistence (**Figure 3**). Furthermore, IL-21-expressing rHBV virions were shown to be infective using HepG2/NTCP cells (**Figure 4**). Collectively, these data suggest rHBV as a valid vector for liver-targeting delivery of IL-21.

In CHB patients, a predominant majority of hepatocytes were already infected with HBV 16, and for IL-21-expressing rHBV virions to be applicable to CHB treatment, the ability to superinfect HBV-infected hepatocyte is a prerequisite. Although the existence of naturally occurring recombinant HBV strains suggests the possibility of coinfection and/or superinfection, superinfection of HBV has not been experimentally studied in detail. In this work, using IL-21-expressing rHBV virions, we demonstrated superinfection by rHBV of HBV-infected cells both *in vitro* and *in vivo* (**Figure 5** and** Figure 6**). Such superinfection not only allows rHBV to engender expression of cargo gene, IL-21 in this case, in HBV-infected cells, but also enables production of rHBV progeny virions using envelope proteins expressed from preexisting wild type HBV genomes (**Figure 5** and** Figure 6**). In addition, the sustained production of rHBV virions and IL-21 in HBV-infected human liver chimeric mice super-infected with IL-21-expressing rHBV through a single injection (**Figure 6**) indicated that *in vivo*, rHBV would be able to persist as long as wild type HBV has not been cleared. In an immunocompetent HBV-infected host, such qualities of IL-21-expressing rHBV would allow continued expression of IL-21 until wild type HBV has been reduced to negligible levels as a result of the effects of IL-21 and/or other co-administered therapeutics. Since IL-21 acts through inducing HBV-specific CD8^+^ T cells in mice 7, and 5c3c-derived rHBV also expresses HBV core antigens (**Figure 1**), it could be envisioned that IL-21-activated HBV-specific cellular immunity (**Figure S7**) would attack and destroy hepatocytes infected with wild type HBV, rHBV, or both. This would effectively constitute an ideal self-removal mechanism that ensures clearance of IL-21-expressing rHBV upon eventual clearance of wild type HBV. Such a mechanism would also partially alleviate the risk associated with possible integration of rHBV sequences into hepatocyte chromosome by targeting hepatocytes with integrated rHBV sequences retaining HBcAg expression.

In summary, results presented here demonstrated that rHBV is a powerful vector for delivering IL-21, and possibly also other HBV-targeting genes, specifically into hepatocytes, including previously HBV-infected hepatocytes. Therapeutic application of such rHBV virions in the context of human chronic HBV infections warrants further studies.

## Materials and Methods

### Plasmids and cell lines

Construction of most of the plasmids used in this work has been previously described. Briefly, replicon plasmid pCMV1.1HBV contains 1.1× over-length wild-type HBV genome under the control of cytomegalovirus (CMV) promoter 12. Recombinant HBV vector 5c3c was derived from a clinically isolated deletion mutant (GenBank Accession Number: FJ518810), and has a total of 384 base pairs of the preS1/polymerase spacer region deleted and two nonsense mutations in downstream S coding sequences 3. Helper plasmid pLMS encodes wild-type L/M/S envelope proteins under the control of Sp1 10. Replicon plasmid BPS (GenBank Accession Number: AF100309.1) contains 1.3× over-length HBV genome on pUC18 backbone 8. To construct IL-21-expressing rHBV, reference mIL-21 (GenBank Accession Number: NM_013693.2) and hIL-21 (GenBank Accession Number: NP_068575.1) cDNA sequences were modified with synonymous mutations where necessary to avoid creating premature termination codons in the overlapping polymerase reading frame (**Figure S2** and **Figure S3**) and inserted into 5c3c in frame with ATG-less preS1 to create 5c3c-mIL-21 and 5c3c-hIL-21, respectively. Mutants with premature termination codons (5c3c-mIL-21*^null^*) or obliterated start codons (5c3c-hIL-21*^null^*) were created through site-directed mutagenesis (**Figure S2** and **Figure S3**).

Human hepatoma cell line Huh-7 was used for replicon analysis and rHBV virion production *in vitro*. HepAD38, which is stably transfected with HBV replicon, was used for wild type HBV virion production. HepG2 cells stably transfected with HBV receptor NTCP (HepG2/NTCP) were used for infection. All cells were cultured and transfected using TurboFect (Invitrogen, China) as previously described 8.

### Mice work

Male specific pathogen-free BALB/c mice aged 6-8 weeks were purchased from Experimental Animal Centre, Fudan University. For analyzing tissue- distribution of infection by AAV, BALB/c mice were injected with 2×10^11^ genome equivalents (geq) of recombinant serotype 8 AAV expressing EGFP (AAV-EGFP, Obio Technology, China) in 200 μL PBS via tail vein. At 1 week post injection (w.p.i.), tissues were collected after sacrifice by cervical dislocation and frozen sections prepared for fluorescent microscopy analysis. For evaluating therapeutic effects of IL-21-expressing rHBV replicon, mice were first injected with 10 μg BPS plasmid DNA via tail vein through hydrodynamic injection (HDI). Four weeks later, mice with persistently positive serum HBsAg were selected and injected with 25 μg 5c3c-mIL-21 or 5c3c-mIL-21*^null^* via HDI, or left untreated, and injected once again 3 weeks later. Serial serum samples and tissues were collected at indicated time points or after sacrifice as previously reported 8. Human liver chimeric mice 17, 18 were purchased from Wuxi App Technology (China).

Mouse procedures were approved by the Animal Ethics Committee of School of Basic Medical Sciences, Fudan University.

### HBV virion production and infection

To obtain wild-type HBV virions produced *in vitro* and *in vivo*, supernatants form HepAD38 cells and sera from BALB/c mice hydrodynamically injected with pCMV1.1HBV at 3 days post injection were concentrated using Amicon Ultra-15 centrifugal filters (Millipore, Billerica, USA). To obtain rHBV virions, rHBV replicon plasmid and helper plasmid pLMS were transfected into Huh-7 cells or injected into BALB/c mice via HDI at 1:1 ratio, and supernatants or sera were concentrated as described above. Infection of HepG2/NTCP cells with HBV was performed using a multiplicity of infection (MOI) of 1000:1 (geq versus cell numbers) as previously reported 8. For superinfection, HepG2/NTCP cells were first infected with wild type HBV at 1000:1 MOI using concentrated sera from pCMV1.1HBV injected mice. At day 5 post infection, concentrated sera from naïve, 5c3c-mIL-21/pLMS or 5c3c-mIL-21*^null^*/pLMS co-injected mice were used to superinfect HepG2/NTCP cells at 1000:1 MOI.

For superinfection in chimeric mice, 2×10^6^ geq of wild type HBV concentrated from pCMV1.1HBV injected mice were injected via tail vein, and 2 weeks later, 2×10^6^ geq of rHBV concentrated from 5c3c-mIL-21/pLMS co-injected mice were injected in the same way.

### HBV antigen and DNA analysis

HBsAg, HBeAg and HBsAb were measured using ELISA (Kehua, China). Mouse and human IL-21 expressed by transfected or infected cells were measured using ELISA (Catolog #EK0797, Boster, China and #RK00041, ABclonal, USA, respectively). Total, wild-type and rHBV DNA in culture medium and mouse serum were measured using quantitative real time PCR. Wild-type and rHBV DNA in tissue samples from sacrificed human liver chimeric mice were analysed using PCR. Primer sequences used are listed in **Table S1**. Capsid-associated HBV DNA and nuclear HBV replicon DNA were extracted from transfected cells and mouse liver cells respectively, and analyzed in Southern blot as previously described 7, 8. HBV DNA in virions and unenveloped capsids were analyzed by immunoprecipitation using anti-PreS1 (sc57761, Santa Cruz) and anti-HBcAg (Dako) antibodies, respectively, prior to HBV DNA analysis as described above.

### Immunofluorescence analysis

HepG2/NTCP cells infected with wild-HBV and/or rHBV virions were subjected to immunofluorescence analysis to detect HBcAg, HBsAg, and mIL-21. Primary antibodies against HBcAg (1:500) and mIL-21 (1:1000) were purchased from Dako (Denmark) and R&D SYSTEMS (USA), respectively. Primary antibody against HBsAg has been previously reported 19. Secondary antibodies Fluor 546 donkey anti-rabbit IgG(H+L) (1:1000), Cy3-conjugated Affinipure rabbit anti-goat IgG(H+L) (1:1000) and FITC-labeled goat anti-human IgG (1:1000) were purchased from Life Technologies (USA), Proteintech (USA) and Beyotime (China), respectively. Cell nuclei were stained with DAPI (4′,6-diamidino-2-phenylindole) (Sangon, China). Fluorescence microscopy was performed using an AMG EVOS fluorescence microscope (Mill Creek, USA).

### T cell immune responses analysis

Mouse splenocytes cultured in RPMI 1640 (Invitrogen) containing 2 mM L-glutamine, 50 U/mL penicillin, and 10% foetal bovine serum (Invitrogen) were stimulated with 15 μg/mL recombinant HBsAg as described 8 or 10 μg/mL recombinant HBcAg (Catalog #hbv-232-a, ProSpec, China) for 72 h. Gamma interferon (IFN-γ) in culture medium was then measured using ELISA (Catalog #D721025, Sango, China).

### Statistical analyses

The levels of serum HBV markers were presented as the mean ± standard errors of the mean (SEM). Statistical significance was calculated using unpaired two-tailed *t*-test. A *P*-value <0.05 was considered to indicate statistical significance in all analyses. Group positivity percentages of serum HBV markers were expressed using Kaplan-Meier plot. GraphPad 6 was used for plotting and statistical tests.

## References

[1] World Health Organization (2019). Hepatitis B. World Health Organization Fact Sheet (Revised 18 July 2019). http://www.who.int/en/news-room/fact-sheets/detail/hepatitis-b.

[2] Lok AS, Zoulim F, Dusheiko G, Ghany MG (2017). Hepatitis B cure: From discovery to regulatory approval. Hepatology.

[3] Seeger C, Mason WS (2015). Molecular biology of hepatitis B virus infection. Virology.

[4] Liang TJ (2009). Hepatitis B: the virus and disease. Hepatology.

[5] Gish RG, Given BD, Lai CL, Locarnini SA, Lau JY, Lewis DL (2015). Chronic hepatitis B: Virology, natural history, current management and a glimpse at future opportunities. Antiviral Res.

[6] Shouval D, Shibolet O (2013). Immunosuppression and HBV reactivation. Semin Liver Dis.

[7] Shen Z, Liu J, Wu J, Zhu Y, Li G, Wang J (2019). IL-21-based therapies induce clearance of hepatitis B virus persistence in mouse models. Theranostics.

[8] Shen Z, Yang H, Yang S, Wang W, Cui X, Zhou X (2017). Hepatitis B virus persistence in mice reveals IL-21 and IL-33 as regulators of viral clearance. Nat Commun.

[9] Leonard WJ, Spolski R (2005). Interleukin-21: a modulator of lymphoid proliferation, apoptosis and differentiation. Nat Rev Immunol.

[10] Spolski R, Leonard WJ (2014). Interleukin-21: a double-edged sword with therapeutic potential. Nat Rev Drug Discov.

[11] Bai W, Cui X, Xie Y, Liu J (2016). Engineering Hepadnaviruses as Reporter-Expressing Vectors: Recent Progress and Future Perspectives. Viruses.

[12] Hong R, Bai W, Zhai J, Liu W, Li X, Zhang J (2013). Novel recombinant hepatitis B virus vectors efficiently deliver protein and RNA encoding genes into primary hepatocytes. J Virol.

[13] Kattenhorn LM, Tipper CH, Stoica L, Geraghty DS, Wright TL, Clark KR (2016). Adeno-Associated Virus Gene Therapy for Liver Disease. Hum Gene Ther.

[14] Pipe S, Leebeek FWG, Ferreira V, Sawyer EK, Pasi J (2019). Clinical Considerations for Capsid Choice in the Development of Liver-Targeted AAV-Based Gene Transfer. Mol Ther Methods Clin Dev.

[15] Ma S, Sun J, Guo Y, Zhang P, Liu Y, Zheng D (2017). Combination of AAV-TRAIL with miR-221-Zip Therapeutic Strategy Overcomes the Resistance to TRAIL Induced Apoptosis in Liver Cancer. Theranostics.

[16] Seeger C, Mason WS (2000). Hepatitis B virus biology. Microbiol Mol Biol Rev.

[17] Lutgehetmann M, Mancke LV, Volz T, Helbig M, Allweiss L, Bornscheuer T (2012). Humanized chimeric uPA mouse model for the study of hepatitis B and D virus interactions and preclinical drug evaluation. Hepatology.

[18] Yang G, Feng J, Liu Y, Zhao M, Yuan Y, Yuan H (2019). HAT1 signaling confers to assembly and epigenetic regulation of HBV cccDNA minichromosome. Theranostics.

[19] Wang W, Sun L, Li T, Ma Y, Li J, Liu Y (2016). A human monoclonal antibody against small envelope protein of hepatitis B virus with potent neutralization effect. MAbs.

