# Supplementary material for: Characterization of IL-21-expressing recombinant hepatitis B virus (HBV) as a therapeutic agent targeting persisting HBV infection
Source: Theranostics. 2020 Apr 25;10(12):5600–12. doi: 10.7150/thno.44715 (PMC7196313; doi:10.7150/thno.44715)
Supplement: Supplementary file 1 — Supplementary figures and tables. [file thnov10p5600s1.pdf]

Supplementary Figures and Tables

Characterization of IL-21-expressing recombinant hepatitis B virus (HBV) as a therapeutic agent targeting persisting HBV infection

Zhongliang Shen, Jingwen Wu, Zixiang Gao, Jingyu Wang, Haoxiang Zhu, Richen Mao, Xuanyi Wang, Jiming Zhang, Youhua Xie, and Jing Liu

LIST

Supplementary Table 1, Supplementary Figure 1-11

Table S1 Primers used for quantitative real time PCR

Target	Primer sequences
Total HBV	5’-AATGCCCCTATCTTATCAACACT-3’<br>5’-GAGATTGAGATCTTCTGCGACG-3’
Wild-type HBV	5’- GATCATCAGTTGGACCCTGCATTCAAA -3’<br>5’- CTGTTGGCACAGTTGTGAGTA -3’
mIL-21 rHBV	5’-TTGCCATGGAGAGGACCCTTGT-3’<br>5’- CTGAAAACAGGCAAAAGCTGCA -3’
Mouse GAPDH	5’-TGCCCAGAACATCATCCCTG-3’<br>5’-TCAGATCCACGACGGACACA-3’
Human GAPDH	5’- GTATGACAACGAATTTGGCTA -3’<br>5’-CCCTCCCCTCTTCAAGGGGT -3’

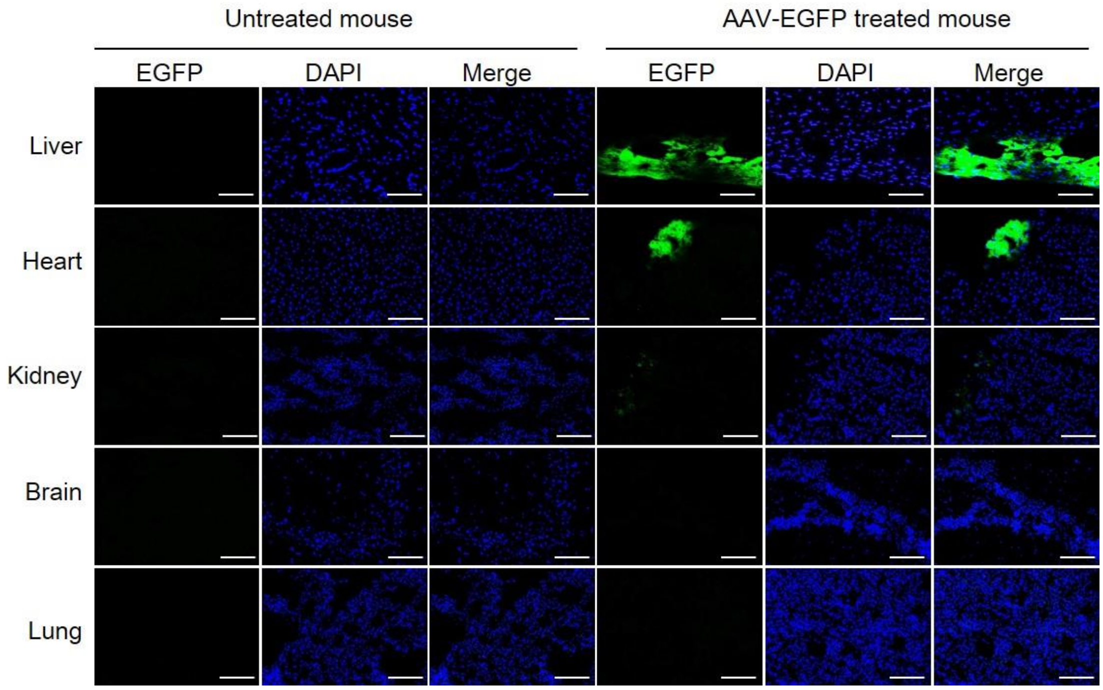

Figure S1. Tissue-distribution of recombinant serotype 8 AAV in mice. Male BALB/c mice aged 6-8 weeks were injected with 2×10^{11} genome equivalents of serotype 8 AAV expressing EGFP (AAV-EGFP) via tail vein or left untreated. One week after infection, tissues were collected from sacrificed mice and subjected to frozen section preparation and DAPI staining. DAPI and EGFP signals were visualized using fluorescence microscopy. Scale bars, 100 μm.

```
ATG GAG AGG ACC CTT GTC TGT CTG GTA GTC ATC TTC TTG GGG ACA
GTG GCC CAT AAA TCA AGC CCC CAA GGG CCA GAT CGC CTC CTG ATT
AGA CTT CGT CAC CTT ATT GAC ATT GTT GAA CAG CTG AAA ATC TAT
GAA AAT GAC TTG GAT CCT GAA CTT CTA TCA GCT CCA CAA GAT GTA
AAG GGG CAC TGT GAG CAT GCA GCT TTT GCC TGT TTT CAG AAG GCC
AAA CTC AAG CCA TCA AAC CCT GGA AAC AAT AAG ACA TTC ATC ATT
GAC CTC GTG GCC CAG CTC AGG AGG AGG CTG CCT GCC AGG AGG GGA
GGA AAG AAA CAG AAG CAC ATA GCT AAA TGC CCT TCC TGT GAT TCG
TAT GAG AAA AGG ACA CCC AAA GAA TTC CTA GAA AGA CTA AAA TGG
CTC CTT CAA AAG ATG ATT CAT CAG CAT CTC TCC TAG
```

Figure S2. Modification of murine IL-21 gene for insertion into 5c3c rHBV vector. Nucleotide sequences of mIL-21 coding sequences according to Gene Bank Accession NM_013693.2 were used for modification. For insertion into 5c3c, synonymous mutations (T to C, red) were introduced at indicated positions to avoid creating premature termination codons in the overlapping polymerase ORF. Nonsense mutations (T to A or C to A, blue) were introduced at indicated positions (blue) to create mIL-21^{null} control sequences.

ATG CAG GGT CAG GAC CGT CAC ATG ATC CGC ATG CGT CAG CTG ATC
GAC ATC GTT GAC CAG CTG AAG AAC TAT GTT AAT GAC CTG GTT CCG
GAG TTT CTG CCG GCA CCT GAA GAC GTA GAA ACG AAC TGC GAA TGG
AGT GCA TTC TCT TGT TTC CAG AAG GCC CAG CTG AAA TCT GCG AAC
ACC GGT AAC AAC GAA CGT ATC ATC AAC GTG TCC ATC AAG AAA CTT
AAG CGC AAA CCG CCG TCT ACC AAC GCT GGT CGC CGT CAG AAA CAC
CGT CTC ACC TGT CCA TCC TGC GAT TCC TAT GAA AAG AAA CCG CCG
AAA GAA TTC CTG GAA CGT TTC AAA TCT CTG CTG CAG AAG ATG ATC
CAC CAG CAC CTG TCT AGC CGC ACT CAC GGT TCT GAG GAC TCC TAA

Figure S3. Modification of human IL-21 gene for insertion into 5c3c rHBV vector. Nucleotide sequences of hIL-21 coding sequences according to Gene Bank Accession NP_068575.1 were used for modification. For insertion into 5c3c, synonymous mutations (T to C, red) were introduced at indicated positions to avoid creating premature termination codons in the overlapping polymerase ORF. In-frame start codons at 1st, 8th and 11th positions were obliterated (T to C, blue) to create hIL-21*null* control sequences.

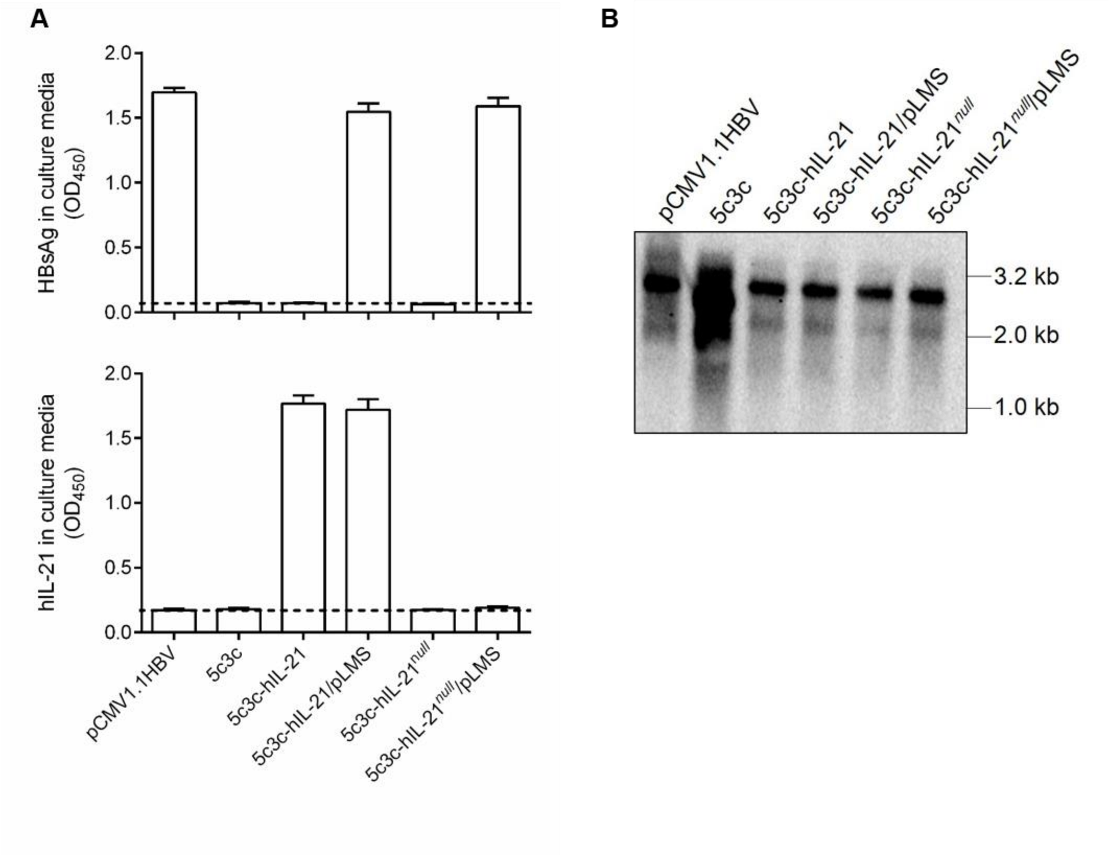

Figure S4. Characterization of 5c3c-derived rHBV expressing human IL-21. (**A**) Supernatants from Huh-7 transfected with indicated plasmids were assayed for HBsAg (top) and hIL-21 (bottom) using ELISA. Group means and SEMs are presented. Dotted lines represent cut-off thresholds. (**B**) Southern blot analysis of intracellular capsid-associated HBV DNA. Transfections were independently repeated with similar results and representative data from one experiment are shown.

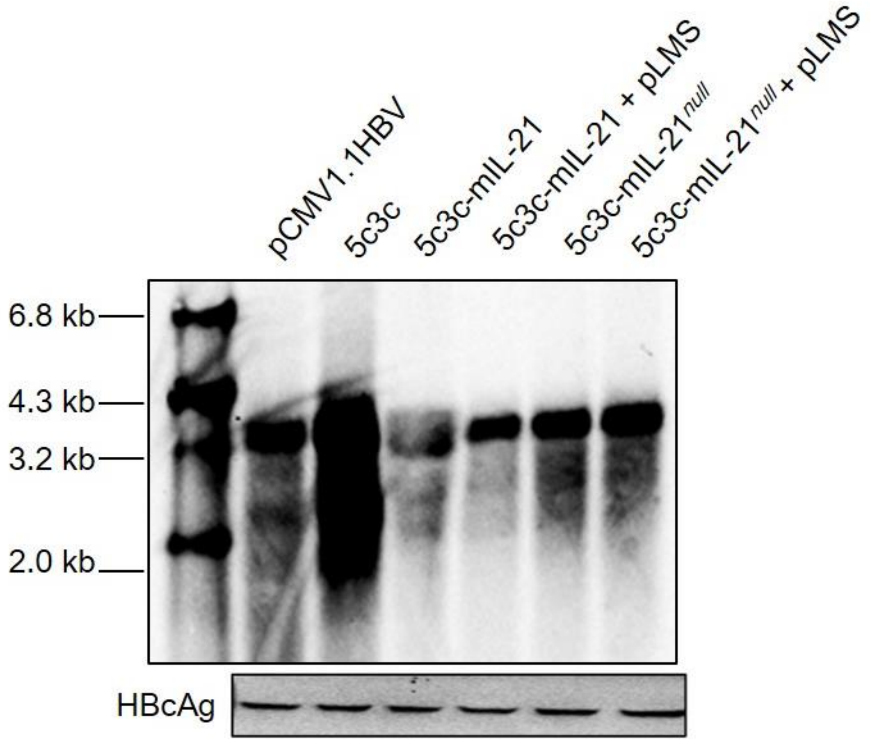

Figure S5. Detection of HBV DNA associated with unenveloped core particles in culture supernatants of transfected cells. Huh-7 cells were transfected with indicated plasmids and culture supernatants were collected 3 days later followed by immunoprecipitation using anti-HBcAg antibody. Captured particles were subjected to DNA extraction and Southern blot analysis using HBV-specific probes (top), and simultaneously analyzed in Western blot using anti-HBcAg antibody to confirm successful pulldown (bottom).

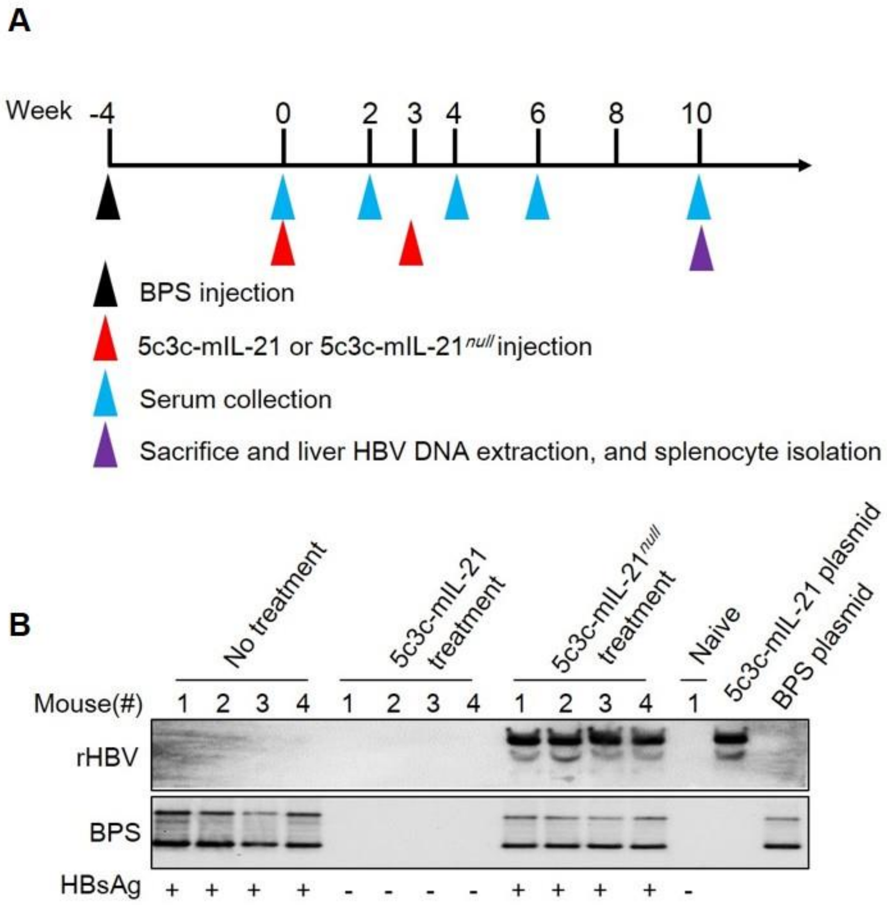

Figure S6. mIL-21-expressing rHBV replicon induces disappearance of intrahepatic viral DNA from HBV persistence mice. (**A**) BALB/c mice injected with BPS replicon plasmid using hydrodynamic injection (HDI) via tail vein that remained persistently positive for serum HBV antigens for 4 weeks were injected using HDI twice with a 3-week interval, with 25 μg of 5c3c-mIL-21 (n = 4) or 5c3c-mIL-21^{null} (n = 4), or left untreated (n = 4). Sera were collected at indicated time points and assayed for HBsAg using ELISA. Mice were sacrificed at 10 w.p.i. with liver and spleen samples taken. All 5c3c-mIL-21 treated mice had remained negative for serum HBsAg for over 4 weeks at sacrifice. (**B**) Nuclear DNA were extracted form liver tissues and subjected to Southern blot using BPS-specific (bottom) and mIL-21-specific (top) probes, with serum HBsAg status indicated below. Splenocytes

were subjected to analysis of HBV specific T cell responses (see **Figure S7**).

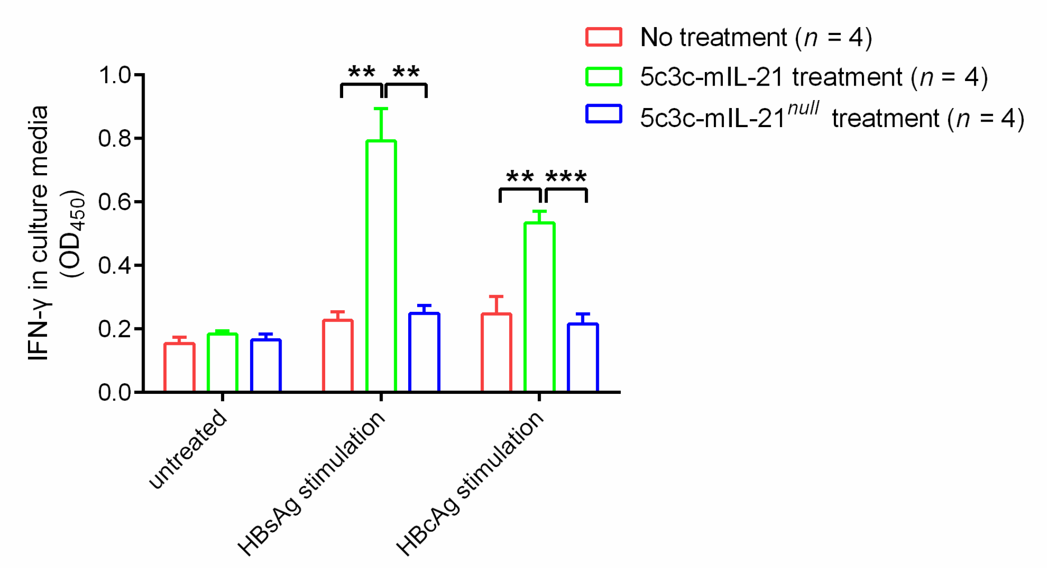

Figure S7. Analysis of HBV-specific spleen T cell responses in HBV persistence mice cured by injection with 5c3c-mIL-21 replicon. Spleen cells from 5c3c-mIL-21 or 5c3c-mIL-21^{null} treated, and untreated BPS HDI mice (see **Figure S6**) were cultured in duplicates and stimulated with recombinant HBsAg or HBcAg proteins. After 3 days of stimulation, IFN-γ in culture media was analyzed in ELISA. Group means and SEMs are presented with group sizes (*n*) indicated. Statistical significance was calculated using unpaired two-tailed t-test. **, $P < 0.01$; ***, $P < 0.001$.

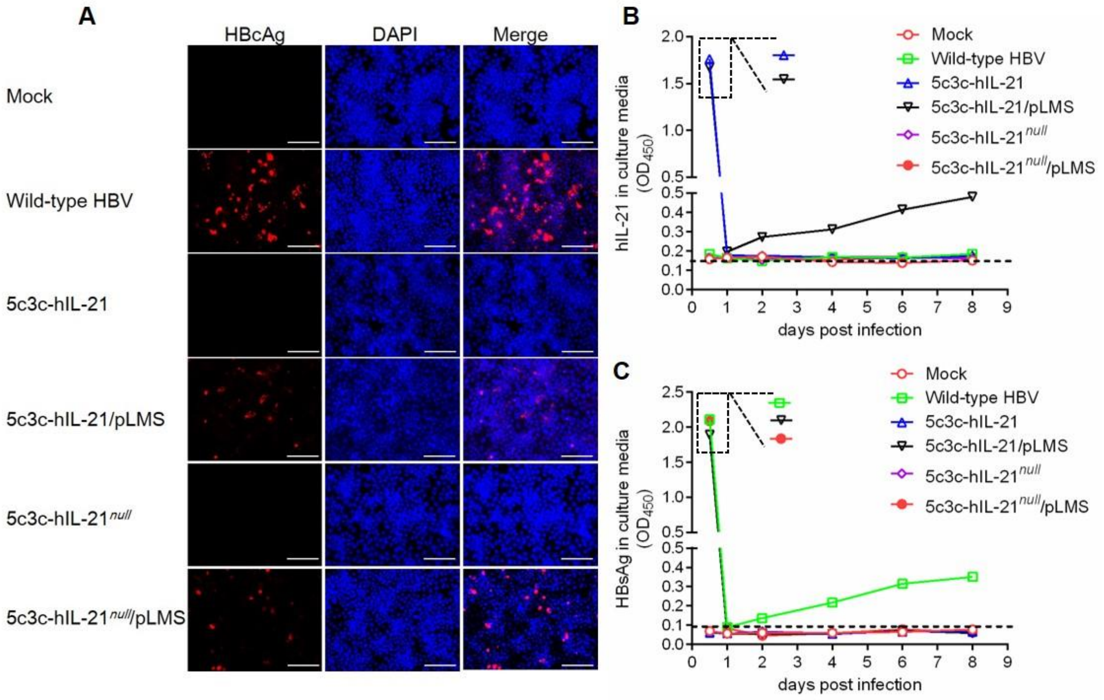

Figure S8. Infection of HepG2/NTCP cells by hIL-21-expressing rHBV virions. Concentrated supernatants from HepAD38 cells, 5c3c-hIL-21/pLMS and 5c3c-hIL-21*null*/pLMS co-transfected Huh-7 cells were used to infect HepG2/NTCP cells. Concentrated supernatants from mock transfection were used as negative control. Concentrated supernatants from 5c3c-hIL-21 and 5c3c-hIL-21*null* mono-transfections, which contained only naked nucleocapsids (see **Figure 1**), were used to exclude the possibility of capsid-mediated transduction. Culture media were changed at day 0.5, 1 and 2 post infection, and then every 2 days. (**A**) On day 8, cells were subjected to DAPI and anti-HBcAg staining and visualized using fluorescence microscopy. Scale bars, 100 μm. hIL-21 (**B**) and HBsAg (**C**) in culture media at indicated time points were measured using ELISA. Dotted lines represent cut-off thresholds. ELISA was performed in duplicates and mean values were used for plotting. Infections were independently repeated with similar results and

representative data from one experiment are shown.

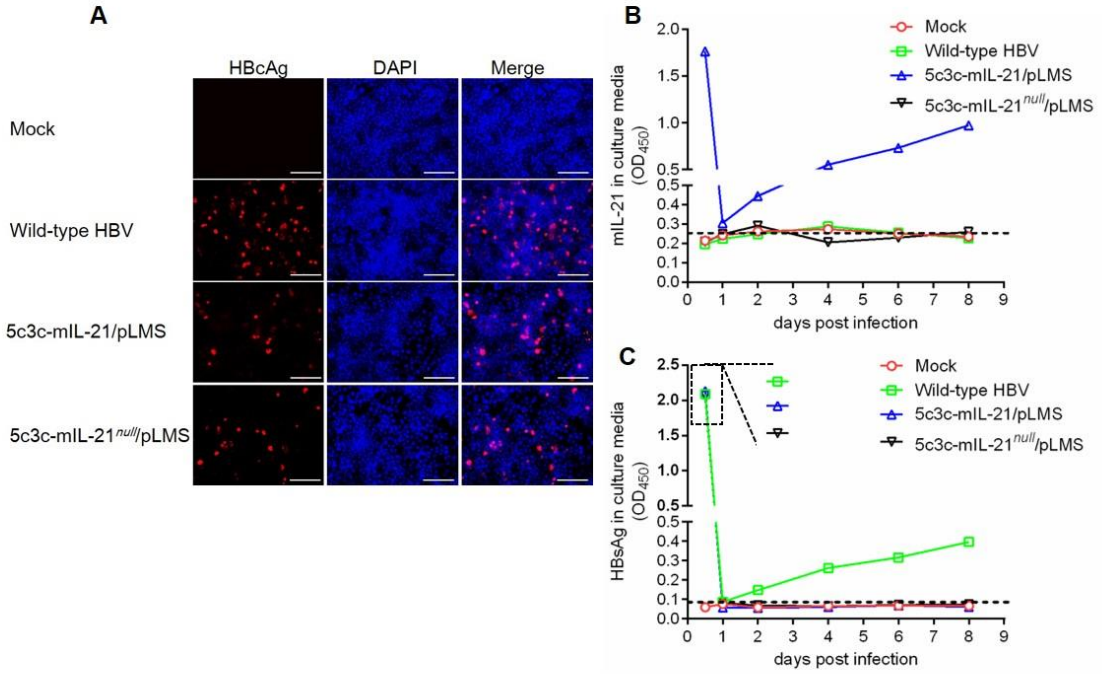

Figure S9. Infection of HepG2/NTCP cells by mIL-21-expressing rHBV virions produced by HDI mice. Concentrated sera from pCMV1.1HBV mono-injected, and 5c3c-hIL-21/pLMS or 5c3c-hIL-21*null*/pLMS co-injected mice were used to infect HepG2/NTCP cells. Culture media were changed at day 0.5, 1 and 2 post infection, and then every 2 days. (**A**) On day 8, cells were subjected to DAPI and anti-HBcAg staining and visualized using fluorescence microscopy. Scale bars, 100 µm. mIL-21 (**B**) and HBsAg (**C**) in culture media at indicated time points were measured using ELISA. Dotted lines represent cut-off thresholds. ELISA was performed in duplicates and mean values were used for plotting. Infections were independently repeated with similar results and representative data from one experiment are shown.

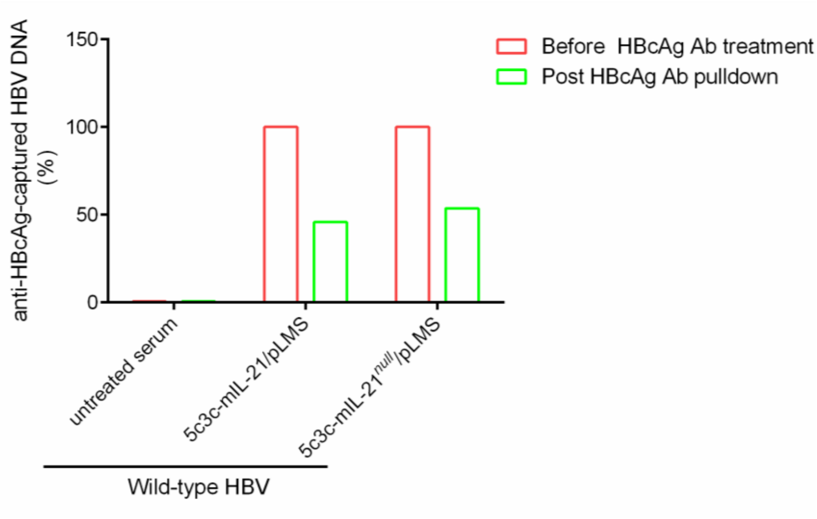

Figure S10. Unenveloped rHBV DNA-containing core particles were produced by rHBV-superinfected HepG2/NTCP cells. Supernatants in **Figure 5** were analyzed using quantitative realtime PCR and primers specific for rHBV before and after capture by anti-HBcAg. Captured rHBV DNA levels were normalized against rHBV DNA levels without antibody pulldown. qrtPCR was performed in triplicates and mean values were used for plotting.

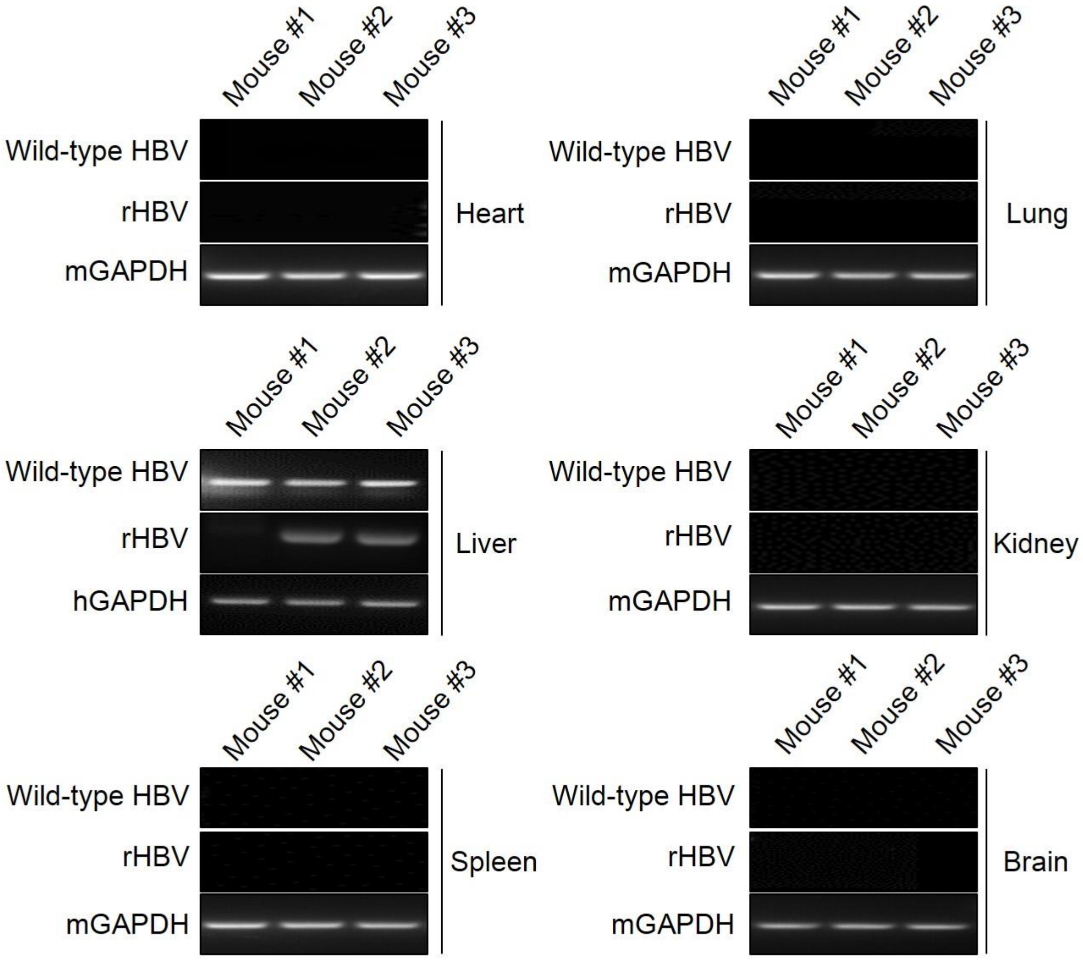

Figure S11. Tissue distribution of HBV and rHBV infection in human liver chimeric mice. Tissue samples taken from mice shown in Figure 6 were subjected to total DNA extraction followed by PCR using primers for wild-type HBV, 5c3c-mIL-21 (rHBV), mouse GAPDH (mGAPDG) or human GAPDH (hGAPDH).
